# Identifying groups of children's social mobility opportunity for public health applications using k-means clustering

**DOI:** 10.1016/j.heliyon.2023.e20250

**Published:** 2023-09-18

**Authors:** Sarah Zelasky, Chantel L. Martin, Christopher Weaver, Lisa K. Baxter, Kristen M. Rappazzo

**Affiliations:** a Oak Ridge Associated Universities at the U.S. Environmental Protection Agency, Chapel Hill, NC, USA; b Department of Epidemiology, Gillings School of Global Public Health, University of North Carolina at Chapel Hill, 135 Dauer Drive, Chapel Hill, NC 27599, USA; c U.S. Environmental Protection Agency, Office of Research and Development, Center for Public Health and Environmental Assessment, Research Triangle Park, Durham, NC, USA

**Keywords:** Clustering, K-means, Socioeconomics, Upward social mobility, Opportunity

## Abstract

**Background:**

The Opportunity Atlas project is a pioneering effort to trace social mobility and adulthood socioeconomic outcomes back to childhood residence. Half of the variation in adulthood socioeconomic outcomes was explainable by neighborhood-level socioeconomic characteristics during childhood. Clustering census tracts by Opportunity Atlas characteristics would allow for further exploration of variance in social mobility. Our objectives here are to identify and describe spatial clustering trends within Opportunity Atlas outcomes.

**Methods:**

We utilized a k-means clustering machine learning approach with four outcome variables (individual income, incarceration rate, employment, and percent of residents living in a neighborhood with low levels of poverty) each given at five parental income levels (1st, 25th, 50th, 75th, and 100th percentiles of the national distribution) to create clusters of census tracts across the contiguous United States (US) and within each Environmental Protection Agency region.

**Results:**

At the national level, the algorithm identified seven distinct clusters; the highest opportunity clusters occurred in the Northern Midwest and Northeast, and the lowest opportunity clusters occurred in rural areas of the Southwest and Southeast. For regional analyses, we identified between five to nine clusters within each region. PCA loadings fluctuate across parental income levels; income and low poverty neighborhood residence explain a substantial amount of variance across all variables, but there are differences in contributions across parental income levels for many components.

**Conclusions:**

Using data from the Opportunity Atlas, we have taken four social mobility opportunity outcome variables each stratified at five parental income levels and created nationwide and EPA region-specific clusters that group together census tracts with similar opportunity profiles. The development of clusters that can serve as a combined index of social mobility opportunity is an important contribution of this work, and this in turn can be employed in future investigations of factors associated with children's social mobility.

## 1 Introduction

In response to the growing body of evidence showing that children's residence has substantial impacts on their adulthood social mobility [[1], [2], [3], [4], [5], [6], [7], [8]], a collaborative project was formed between researchers from Harvard University, Brown University, and the United States (US) Census group created and published the Opportunity Atlas [9,10], a pioneering effort that traces adulthood socioeconomic outcomes (e.g., income, marital status, employment, etc) of 20.5 million US children to their childhood census tract of residence. In their analysis, the Opportunity Atlas researchers considered effect modifiers such as race, sex, and parental income and concluded that observable neighborhood characteristics explained 50% of tract-level variation in children's outcomes [9]. This leaves the potential to explore other aspects of the data that could explain the remaining variability, including highlighting spatial trends in outcomes or investigating the explanatory variables for these differences in outcomes. In addition, it opens the question of how the Opportunity Atlas can be leveraged to support public health decision making.

Our understanding of how experiences, exposures, and contexts cumulatively impact and influence health and well-being is continuously developing, and as part of this process there are prospects for examining factors that intersect social, economic and environmental domains. Children's opportunity for upward social mobility is a potential driver of health and well-being, as it will likely influence access to care, ability to respond to adverse exposures, in addition to general resource availability.

The Opportunity Atlas could be a potential data source for researchers; however, the complexity of the data and potential variation across US regions make working with the full datasets an enormous challenge. The Opportunity Atlas is not a composite indicator of children's social mobility, but a collection of many different variables at different parental incomes levels with different levels of national coverage and geographic resolution, along with some variables that are only applicable to specific types of individuals. Across this variety of data, dimension reduction techniques can be used to create a single composite indicator of children's social mobility from the Opportunity Atlas variables.

Clustering techniques have been used for dimension reduction across many areas of research, including epidemiology [11], environmental sciences [12]. These techniques are used to identify subgroupings of data based on correlated features within the data, and these subgroupings can then be used in further analyses. Context of the outcomes is an important factor to consider; for example, opportunity for social mobility to children born in cities may be vastly different depending on underlying regional economic structure [9]. Or opportunity for social mobility may vary between regions due to different regions’ major industries, job availability, or capacity for policies that support opportunity. Therefore, it is important to examine results not only across the US but also for subsets of geographic areas.

Our objectives here are to identify and describe spatial clustering trends within Opportunity Atlas outcomes, and to develop a composite index of spatially distinct clusters for future explorations of predictors of children's social mobility. To do this, we selected four main outcomes (representative of all Opportunity Atlas outcomes) at five parental income levels of the national distribution and used these as inputs in a k-means clustering algorithm such that census tracts with similar magnitudes of variables were clustered together. Because of potential variability across areas because of underlying structural differences, clustering was also performed individually for each of the 10 US Environmental Protection Agency (EPA) Regions to provide greater detail on which census tracts were most similar.

## 2 Methods

### 2.1 Overview

**Fig. 1:**
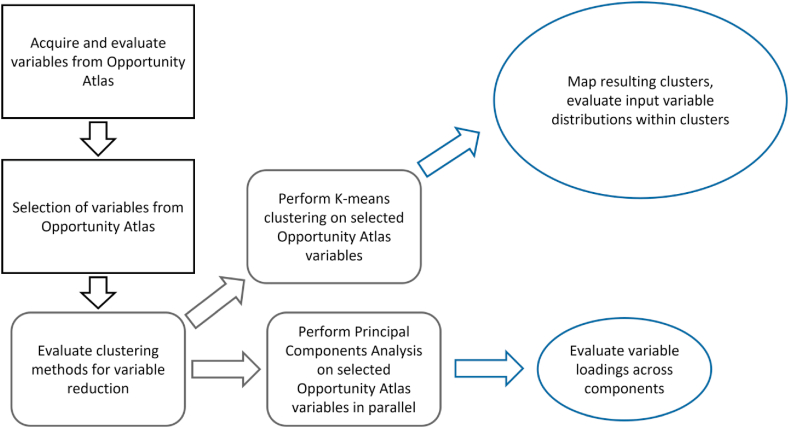
methods process flow for spatial clustering of Opportunity Atlas outcome variables.

We created social mobility clusters of US census tracts using the US Opportunity Atlas dataset, which estimates social mobility outcomes for those who grew up within specific census tracts based on 1978–1983 US birth cohorts [9]. Due to the numerous social mobility outcome variables, we limited our analysis to four “key” outcome variables of individual income (Inc), incarceration rate (Jail), employment, (Work), and percent of residents now living in a neighborhood with levels of poverty <10% (LowPov) that were selected by criteria regarding individual focus, general applicability, and perception of outcome, as described in more detail below. Clustering at the nationwide-level and at the EPA region-level was performed in RStudio 4.0.0 [[13], [14]] using each of the four variables at five parental income levels as the inputs. For each clustering scenario, maps were produced in QGIS 3.7 [15] that displayed differences in the four key variables as well as the assigned clusterings across the US census tracts. Clusters in each scenario were ranked from “best” to “worst” opportunity gradients for residents. A Principal Components Analysis (PCA) was also performed to better understand how social mobility outcomes load together across parental income categories, and which variables contribute the most to specific components. The methods process flow is shown in Fig. 1.

### 2.2 US opportunity atlas and key variable selection

**Fig. 2:**
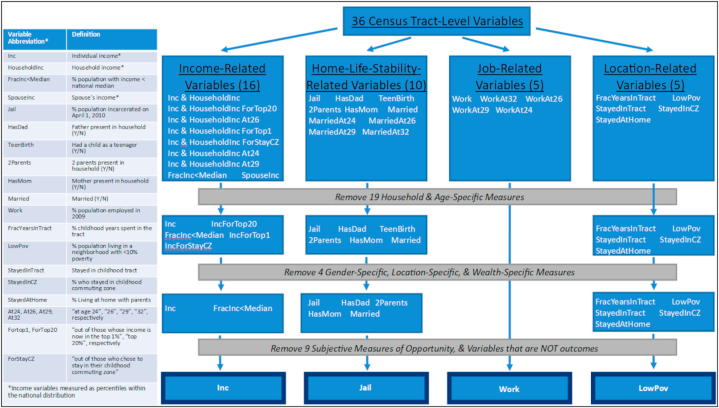
Key Variable Selection Process. Inc: individual income measured as percentile of the national income distribution; Jail: incarceration rate measured as percent of residents who are incarcerated; Work: employment measured as percent of residents who are employed; LowPov: low-poverty neighborhood residence measured as percent of residents who live in a neighborhood with <10% poverty.

Social mobility outcomes were gathered from the US Opportunity Atlas dataset (opportunityatlas.org) [10]. Within this database, 36 variables (each stratified by eight different parental income percentiles) were reported in 2018 at the census tract-level for the 71,215 US census tracts. Due to the similarity of many variables within the Opportunity Atlas, we organized the variables into four broad categories – income-, home life stability-, occupation-, and location-related variables – and selected a “key” variable to represent each of the categories. Additionally, only five of the parental income percentiles were used (1st, 25th, 50th, 75th, and 100th), as they fully represented the income distribution, with the 10th percentile, 90th percentile, and the percentile corresponding with the “average income” being excluded due to the redundancy. The criteria for “key” variables were as follows: 1) each key variable represents the success of the individual (not the household) and represents average adulthood outcomes (not outcomes for individuals of a specific age), 2) each key variable applies to all people (not exclusively a statistic that applies to women or the wealthy at the top 1% and top 20%), and 3) each key variable represents an objective *successful outcome* (neither a variable which may be perceived as desirable for some and not for others nor a variable that is not an outcome). The variable selection process resulted in individual income (abbrev: Inc; measured as percentile of the national income distribution), incarceration rate (abbrev: Jail; measured as percent of residents who are incarcerated), employment (abbrev: Work; measured as percent of residents who are employed), and low-poverty neighborhood residence (abbrev: LowPov; measured as percent of residents who live in a neighborhood with <10% poverty) being chosen as the four key variables extracted from the Opportunity Atlas (Fig. 2). Each of these variables is an outcome measured on April 1, 2010 when the most recent census data at the time was collected, and rates were calculated as the prevalence of an outcome divided by the total number of cohort members in a tract. It is also important to note that the selected variables are adulthood outcomes that resulted from the holistic environment in which an individual was raised. So, for a variable such as incarceration rate, this measure not only considers opportunity factors that lead to crime, but also the likelihood of local law enforcement to incarcerate individuals.

### 2.3 Clustering

The k-means algorithm was chosen for clustering because of its high-performance capacity with large datasets and its ability to create clusters that group together substantial portions of the data (>5%) and ignore outliers [16]. Clusters of all US census tracts were created at the national level, and smaller clusters were also created within each EPA region using the same four key variables and cluster size requirements; this was done to account for potential geographic variation in underlying mobility structures, such as regional differences in cost of living, and to identify which census tracts have higher opportunity within their regional economy. For each clustering scenario, multiple clustering solutions were created in order to produce the maximum number of clusters for each scenario that still contained n>5% of the data in each cluster. Clusters and their key variable data were exported from R Studio 1.4 for further statistical analysis and mapping.

### 2.4 Mapping & analysis

Using QGIS 3.7 Software [15], the key variables and clustering results were joined to a shapefile containing the U.S. census tract geographic boundary data identified by FIPS (Federal Information Processing System) codes [17]. Each census tract was classified as urban, suburban, or rural based on Rural Urban Commuting Area Codes (RUCA) Codes (1–3: Urban (metropolitan); 4–6: Suburban (micropolitan); 7–10: Rural (small town and rural)) [18]. Maps were then created to visualize the key variables and clustering scenarios at the census tract-level across the continental U.S., and tables of summary statistics for the clusters were calculated to provide context for the maps. To simplify our results, the tables only include the mean outcomes for individuals who had parental income at the 25th percentile of the national distribution while the maps present clusters that were created using data from individuals of all parental income levels. By focusing on the 25th percentile of income in the tables, we can see what the opportunity would be like for someone from a lower (but not the lowest) socioeconomic background to rise up to a higher socioeconomic status as an adult.

A Principal Components Analysis (PCA) was performed to better understand which key variables contributed most to the creation of the clusters. For this analysis components with Eigenvalues above one were retained.

### 2.5 Sensitivity analyses

To better compare distributions of input variables, we standardized them by calculating z-scores for the data. As the variable distributions might have influenced weights assigned by the algorithm, we also used the standardized versions in a sensitivity analysis with the k-means clustering. We also considered that factors such as race or degree of rurality could confound or modify the relationship between the four key variables and cluster assignment; thus, we created clusters with race-stratified and RUCA-stratified data for context. Because RUCA 1 was the most common classification among the census tracts, we performed an additional clustering scenario in which we clustered only the census tracts with RUCA 1 designation.

## 3 Results

Input variable distributions at the 25th parental income percentile are shown in Supplemental Fig. S1, while correlations between variables at each income level are shown in Supplemental Table S1. Income and employment have relatively similar distributions across the contiguous US with higher incomes and employment rates in the Northeastern US and the Midwest. There were higher incarceration rates in the Southeastern US generally, but with the highest rates associated with individual census tracts located in the Western US. The highest spatial disparities are seen for the low-poverty neighborhood variable in which the majority of Southeast US tracts produced individuals who live in neighborhoods with >10% poverty levels, while the rest of the country has a more heterogeneous distribution of this variable. It is important to note that metropolitan areas appear to consistently have census tracts that produce individuals who live in neighborhoods with <10% poverty levels.

## 4 Clustering of census tracts

**Fig. 3:**
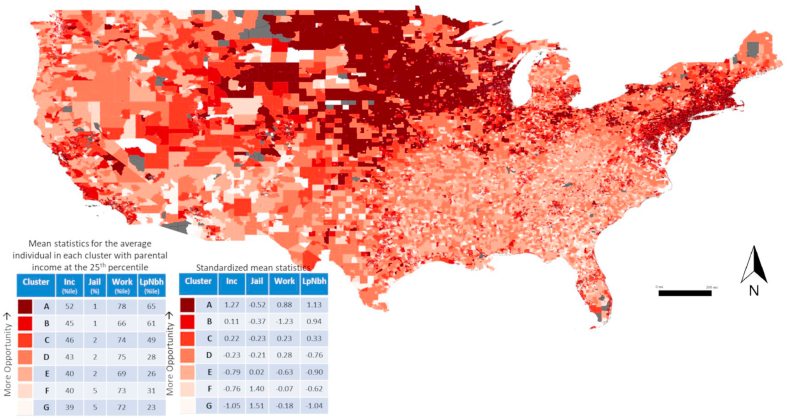
Distribution of clusters across the United States, with mean statistics at the 25th parental income level for each cluster. Inc: individual income measured as percentile of the national income distribution; Jail: incarceration rate measured as percent of residents who are incarcerated; Work: employment measured as percent of residents who are employed; LowPov: low-poverty neighborhood residence measured as percent of residents who live in a neighborhood with <10% poverty.

**Fig. 4:**
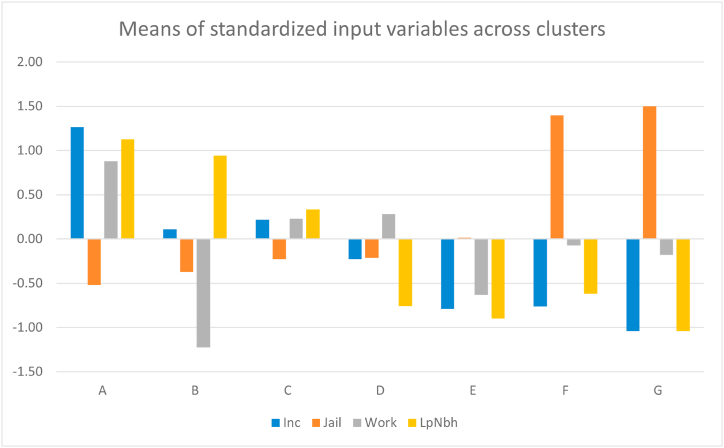
Distributions of standardized mean statistics at the 25th parental income level for each cluster, from most to least opportunity for children's social mobility. Inc: individual income measured as percentile of the national income distribution; Jail: incarceration rate measured as percent of residents who are incarcerated; Work: employment measured as percent of residents who are employed; LowPov: low-poverty neighborhood residence measured as percent of residents who live in a neighborhood with <10% poverty.

The clustering analysis at the full US-level produced seven distinct social mobility groupings (Fig. 3). Clusters with the highest opportunity are typically located in Northeastern metropolitan regions, the Midwest, and are relatively heterogeneous throughout the rest of the contiguous US. Using standardized input variables did not have a major influence on the weights or spatial clustering patterns (Supplemental Fig. S2). At the 25th parental income level the highest opportunity cluster (“A”) had higher income levels, lower incarceration, high – though not the highest – area level employment, and high low poverty neighborhood residence; the worst opportunity cluster (“G”) had a similar but opposite distribution of input variables, while distributions varied across other clusters (Fig. 4).

**Fig. 5:**
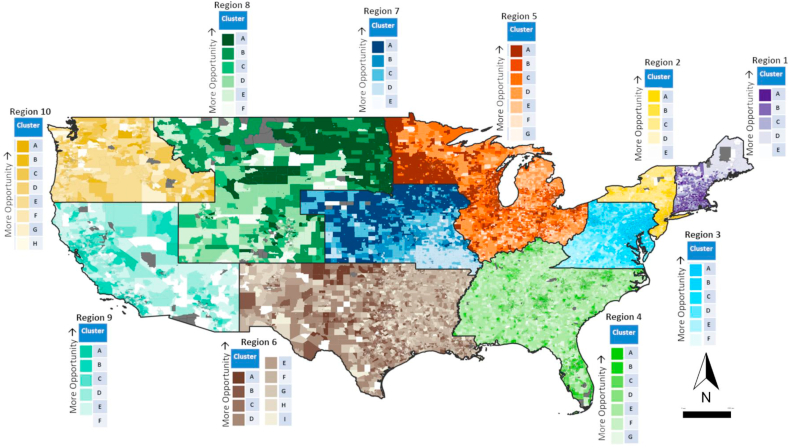
Distribution of clusters across the EPA regions in the United States.

**Fig. 6:**
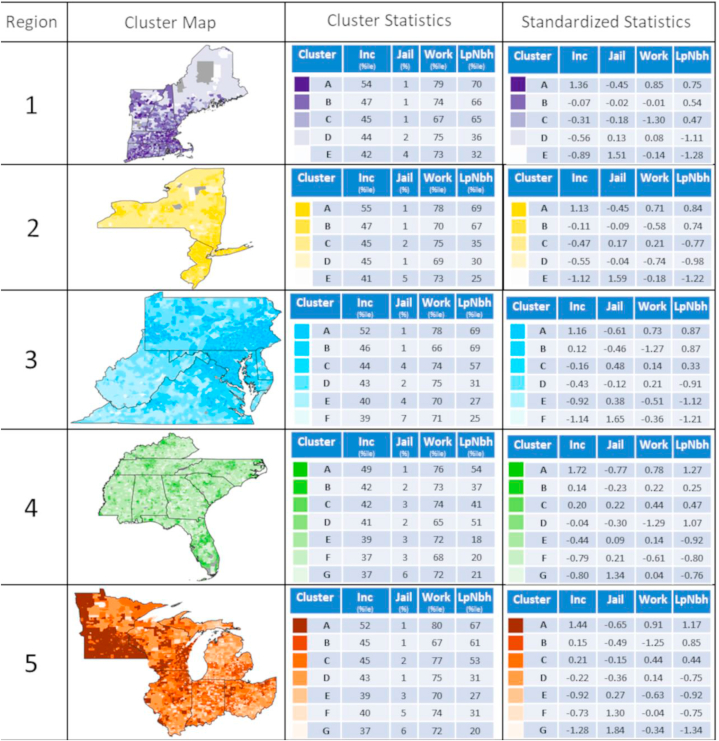

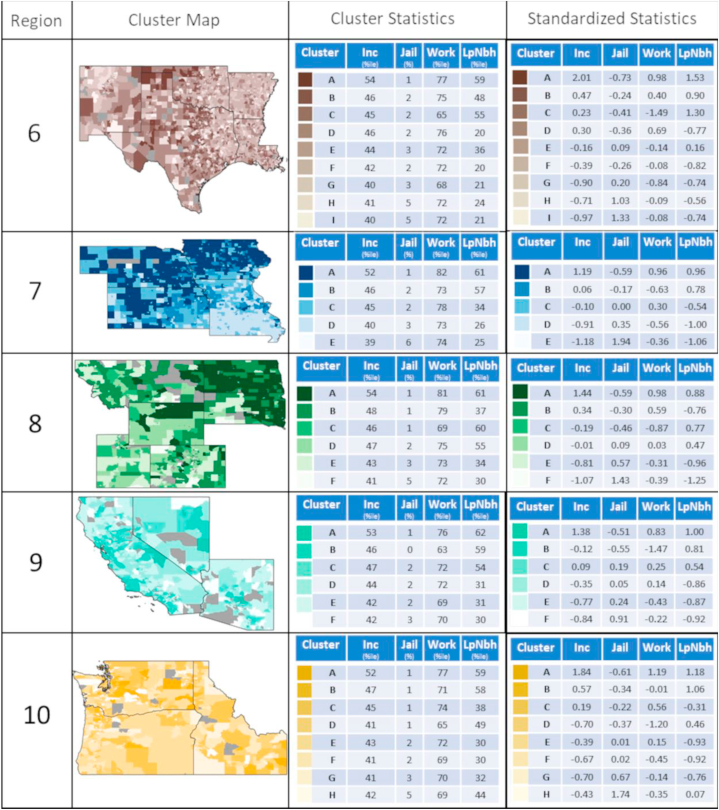
A Social mobility opportunity clusters for EPA Regions 1–5 and their corresponding mean input variable statistics. Note: Statistics represent the mean outcomes for individuals who had parental income at the 25th percentile of the national distribution while the maps show clusters derived from all four key variables at all five parental income levels. Inc: individual income measured as percentile of the national income distribution; Jail: incarceration rate measured as percent of residents who are incarcerated; Work: employment measured as percent of residents who are employed; LowPov: low-poverty neighborhood residence measured as percent of residents who live in a neighborhood with <10% poverty Fig. 6b: Social mobility opportunity clusters for EPA Regions 6–10 and their corresponding mean input variable statistics. Note: Statistics represent the mean outcomes for individuals who had parental income at the 25th percentile of the national distribution while the maps show clusters derived from all four key variables at all five parental income levels. Inc: individual income measured as percentile of the national income distribution; Jail: incarceration rate measured as percent of residents who are incarcerated; Work: employment measured as percent of residents who are employed; LowPov: low-poverty neighborhood residence measured as percent of residents who live in a neighborhood with <10% poverty.

Clustering was performed separately within each EPA region to explore variation across lower geographical aggregates and to illustrate the potential for regional differences in areas of different underlying social and economic environments. The number of clusters for each region was decided based on the maximum number of clusters possible that still allowed for each cluster to have >5% of the total number of census tracts within a region. Cluster distributions for each region are shown in a national map in Fig. 5, with region specific maps and corresponding mean input variable statistics are presented in Fig. 6a and b. The number of distinct social mobility opportunity clusters varied across regions from five to ten. On a nationwide-scale, clusters appeared highly dependent on regional location as well as differences in urbanicity. When broken down into EPA region-specific analyses; however, clusters appeared more heterogeneous across each region.

### 4.1 Principal component analysis

A principal component analysis was performed in order to understand how the input variables across all included parental incomes vary together, and how much overall variance they explain as a whole. In PCA, six components were chosen as the minimum number of components that explained at least 90% of the clustering variance. Within each component, variable loadings are shown in Table 1.

**Table 1:** Results from Principal Component Analysis, including proportion of variance and individual variable loadings for each retained component.

Input var	Comp 1	Comp 2	Comp 3	Comp 4	Comp 5	Comp 6
**Proportion of Variance (%)**	38.6	16.8	13.0	11.2	9.4	5.6
Individual income						
Inc 1	0.242	−0.126	0.196	0.257	0.105	−0.326
Inc 25	0.296	−0.048	0.128	0.174	0.028	−0.390
Inc 50	0.318	0.051	0.029	0.052	−0.068	−0.411
Inc 75	0.284	0.155	−0.090	−0.099	−0.165	−0.358
Inc 100	0.169	0.237	−0.206	−0.250	−0.236	−0.201
Incarcerated residents						
Jail 1	−0.134	0.144	−0.016	−0.313	0.527	−0.217
Jail 25	−0.179	0.122	0.164	−0.357	0.394	−0.225
Jail 50	−0.194	0.039	0.424	−0.305	0.015	−0.157
Jail 75	−0.142	−0.039	0.489	−0.163	−0.288	−0.051
Jail 100	−0.092	−0.081	0.462	−0.056	−0.432	0.019
Working residents						
Work 1	0.072	0.110	0.323	0.393	0.297	0.112
Work 25	0.140	0.302	0.288	0.280	0.210	0.175
Work 50	0.176	0.446	0.118	0.006	0.002	0.191
Work 75	0.141	0.401	−0.066	−0.224	−0.172	0.134
Work 100	0.172	0.449	0.073	−0.055	−0.044	0.182
Low Poverty Neighborhood						
LowPov1	0.290	−0.206	0.077	−0.145	0.065	0.101
LowPov25	0.299	−0.208	0.076	−0.169	0.074	0.129
LowPov50	0.303	−0.204	0.072	−0.200	0.084	0.169
LowPov75	0.292	−0.189	0.064	−0.224	0.092	0.204
LowPov100	0.266	−0.163	0.053	−0.236	0.094	0.228

Component 1 explained 39% of observed variance among the Opportunity Atlas variables, with high positive loadings for income and low poverty neighborhood variables, incarceration variables had low negative loadings and work variables had low positive loadings. In component 2 work variables loaded highly positively, with moderate negative loadings for low poverty neighborhood variables. For component 3, incarceration variables above the 50th parental income percentile loaded highly positively along with work variables below the 25th parental income percentile; income also contributed to this component with positive loadings for variables below the 25th parental income percentile, and a negative loading for the highest parental income percentile. Component 4 had moderate loadings for lower parental incomes incarceration (negative), income and work (positive), and generally negative loadings for living in a low poverty neighborhood. Component 5 had a high positive and high negative loading for the lowest and highest parental income levels (respectively) with incarceration, along with moderate positive loadings for work variables at the lowest parental income levels. Finally, component 6 explained 5.6% of observed variance among the Opportunity Atlas variables, with high negative loadings for income, moderate negative loadings for incarceration, and moderate positive loadings for work and low poverty neighborhood variables. A scree plot and biplot of the components and their variables can be found in Supplemental Info (Figs. S2 and S3).

### 4.2 Sensitivity analyses

Race and ethnicity specific clusters, along with descriptions, are provided in supplementary materials (Supplemental Fig. S5). Briefly, clustering using data for non-Hispanic White individuals is similar to the overall clustering results, while those for other races and ethnicities vary due to underlying geographic distributions and sparsity. In urbanicity analyses, urban census tracts tended to be in both the best and worst opportunity clusters, while mid-level opportunity clusters had more rural census tracts. For RUCA 1 census tracts, the highest opportunity clusters were located in Northeastern metropolitan regions, the Midwest, and were relatively heterogeneous throughout the rest of the contiguous US, with the addition of some high opportunity areas along the West Coast (Supplemental Fig. S6).

## 5 Discussion

In this work, we clustered similar census tracts to achieve a composite index of children's social mobility opportunity and ranked the clusters from best to worst adulthood socioeconomic outcomes, identifying gradations in that opportunity. Previous works have demonstrated this type of clustering and gradation to be useful in other social and environmental research contexts [[19], [20], [21], [22]]. The k-means clustering of US census tracts based on income, incarceration, employment, and residence variables from the Opportunity Atlas yielded seven distinct clusters at the national level, and between five and nine clusters for each of the EPA regions. The national-level clusters were distributed somewhat regionally with the highest opportunity clusters occurring in the Northern Midwest and Northeastern US, and the lowest opportunity clusters occurred in rural areas of the Southwest and Southeast US. For clusterings within each EPA region, clusters were spatially distributed more heterogeneously although it appears that clusters of high opportunity were almost always present in larger metropolitan areas. Results from PCA show how input factors vary together across parental income levels. In particular that while income and residence in a low poverty neighborhood explain a substantial amount of variance across all included variables, there are differences in contributions across parental income levels for many of the relevant components. This highlights the importance of considering these interactions between parental income and social mobility outcomes in creating a composite index.

Within the initial Opportunity Atlas analysis of factors, researchers identified neighborhood employment levels, mean household income, and proportion of college graduates as factors contributing to childhood social mobility [9]. Though these factors do not explain all variability in childhood social mobility, we can expect that they will be large contributors to the clusters developed and described here.

The differences in clusters across the US could be due to factors such as different economies, labor markets, cultures, or historical areas of discrimination and opportunity, while the differences in spatial trends within each EPA likely reflect more local patterns of these factors. Upon further analysis of these 10.13039/100000139EPA region clusterings, it becomes apparent that some clusterings correlate with RUCA Codes (Regions 1, 2, 3, 4) in which metropolitan areas had higher opportunity level clusters, which could indicate better access to resources or support that improve opportunity within those metropolitan areas as opposed to in more rural areas. Some clusterings correlate with state-lines (Regions 6, 7, 8) in which mid-West states with large-scale income-generating industries had higher opportunity level clusters. And some clusterings are heterogeneously spread out without any obvious explanation as to why they are spatially distributed as such (Regions 5, 9, 10). However, even when spread heterogeneously across a region, most tracts within the top clusters had close proximity to major metropolitan areas. When we compare how the national clusters and the regional EPA clusters were derived, we see some level of consistency between the clusters with the most opportunity and the clusters with the least opportunity. On one end, there are census tracts that produced individuals with the highest income, lowest incarceration rates, highest employment, and least impoverished neighborhoods, and at the other end tracts that produced individuals with low income, high incarceration rates, low employment, and high poverty. The middle clusters show some consistency across the regions although each has varying magnitudes of the key opportunity variables. While RUCA codes and state-line boundaries might be able to explain some of the variability in cluster distributions, these components still do not identify the root causes of opportunity inequality. There are many root-cause factors that might make a rural census tract different than an urban census tract in terms of opportunity – educational attainment [23], proximity to jobs [24], or childhood environmental exposures to air and water toxins [25].

There are studies that have begun to explore linkages between the opportunity atlas and environmental exposures or health outcomes [23,[26], [27], [28], [29], [30]]. However, these studies examine associations with single Opportunity Atlas variables due to the constraints of the data as provided. In follow-up analyses, we aim to take our newly derived clusters and assess how they are associated with environmental exposure explanatory variables. Other public health researchers can also utilize these clusters similarly to link them to other explanatory variables.

The clustering performed in this paper is merely one of many ways in which we can group together and better understand multiple opportunity outcome variables. As such, we must be sure to contextualize the clusters when interpreting their spatial trends as indicators of opportunity. The clusters were made by an algorithm that identified differences between four variables each stratified at five income levels. However, other clustering algorithms with different inputs or clustering rules could have produced different results. We chose a k-means clustering algorithm because it was robust to outliers and created fairly equal sized cluster groups that could be interpreted and described in the context of our research. The national ranking of census tracts from 1 to 7 in terms of “opportunity level” is easy to understand and useful in future public health analyses when seeking to associate other geographic variables to holistic opportunity.

There are also limitations to consider in our analysis. There were not enough data within race-specific strata of the Opportunity Atlas to include race as a variable in our clustering, but future analyses would benefit from including this variable if possible since racialization and racism are important contributors to the potential for social mobility [9]. While standardized variable statistics are sometimes shown in the results, our algorithm created clusters using unstandardized variables that might have effectively given differing weights to each variable. However, we performed a sensitivity analysis in which we clustered the standardized variables to ensure that weighting wasn't biasing the clustering results, and we did not see major differences between the clustering distributions.

## 6 Conclusion

Using data from the Opportunity Atlas, we have taken four social mobility opportunity outcome variables each stratified at five parental income levels and created nationwide and EPA region-specific clusters that group together census tracts with similar opportunity profiles. The development of clusters that can serve as a combined index of social mobility opportunity is an important contribution of this work, and this in turn can be employed in future investigations of factors associated with children's social mobility. Variation of social mobility opportunity across parental income levels is not unexpected, however, how different factors vary across those income levels can provide both context for social mobility and prospects for developing strategic resource deployment to improve future social mobility. Similarly, identifying that area-level aggregation is a strong influencer of how an index of children's social mobility opportunity can inform decision making to better address the needs of those in areas of interest. Future work will explore potential contributors to children's social mobility beyond socio-economic factors, with the expectation that identification of relevant elements could inform targeted actions to improve health and well-being outcomes for future generations.

## Disclaimer

The research described in this article has been reviewed by the Center for Public Health and Environmental Assessment, US EPA, and approved for publication. Approval does not signify that the contents necessarily reflect the views and policies of the Agency, nor does the mention of trade names of commercial products constitute endorsement or recommendation for use.

## Author contribution statement

Kristen Rappazzo; Lisa K Baxter: conceived and designed the experiments; analyzed and interpreted the data and wrote the paper.

Sarah Zelasky: performed the experiments; analyzed and interpreted the data and wrote the paper.

Chantel L Martin; Christopher Weaver: analyzed and interpreted the data and wrote the paper.

## Data availability statement

Data included in article/supp. material/referenced in article.

Supplementary content related to this article has been published online at [URL].

## Declaration of competing interest

The authors declare that they have no known competing financial interests or personal relationships that could have appeared to influence the work reported in this paper.
