# Supplementary material for: Identifying groups of children's social mobility opportunity for public health applications using k-means clustering
Source: Heliyon. 2023 Sep 18;9(9):e20250. doi: 10.1016/j.heliyon.2023.e20250 (PMC10560027; doi:10.1016/j.heliyon.2023.e20250)
Supplement: Multimedia component 3 [file mmc3.docx]

**Identifying groups of children’s social mobility opportunity for public health applications**

**using k-means clustering**

Sarah Zelasky^1^, Chantel L Martin^2^, Christopher Weaver^3^, Lisa P Baxter^3^*, Kristen M Rappazzo^3^*†

**Supplementary Materials**

Contents

[Supplemental Figure S1: Key Variable distributions for individuals who had parental income at the 25th percentile level 2](#_Toc141222994)

[Supplemental Table S1: Correlations across selected opportunity atlas variables at five parental income levels. 3](#_Toc141222995)

[Supplemental Figure S2. Sensitivity Analysis: Six US-wide clusters from standardized Opportunity Atlas variables 4](#_Toc141222996)

[Supplemental Figure S3: Screeplot of variance of the dataset explained by each component in PCA. 5](#_Toc141222997)

[Supplemental Figure S4: Biplot of each variable and its relationship to Components 1 & 2 during PCA. 6](#_Toc141222998)

[Supplemental Figure S5. Race/ethnicity-specific clusterings derived from the four Opportunity Atlas key variables at five parental income levels. 7](#_Toc141222999)

[Supplemental Figure S6. US-wide clusters among RUCA 1-designated census tracts and their corresponding statistics derived from the Opportunity Atlas key variables. 8](#_Toc141223000)


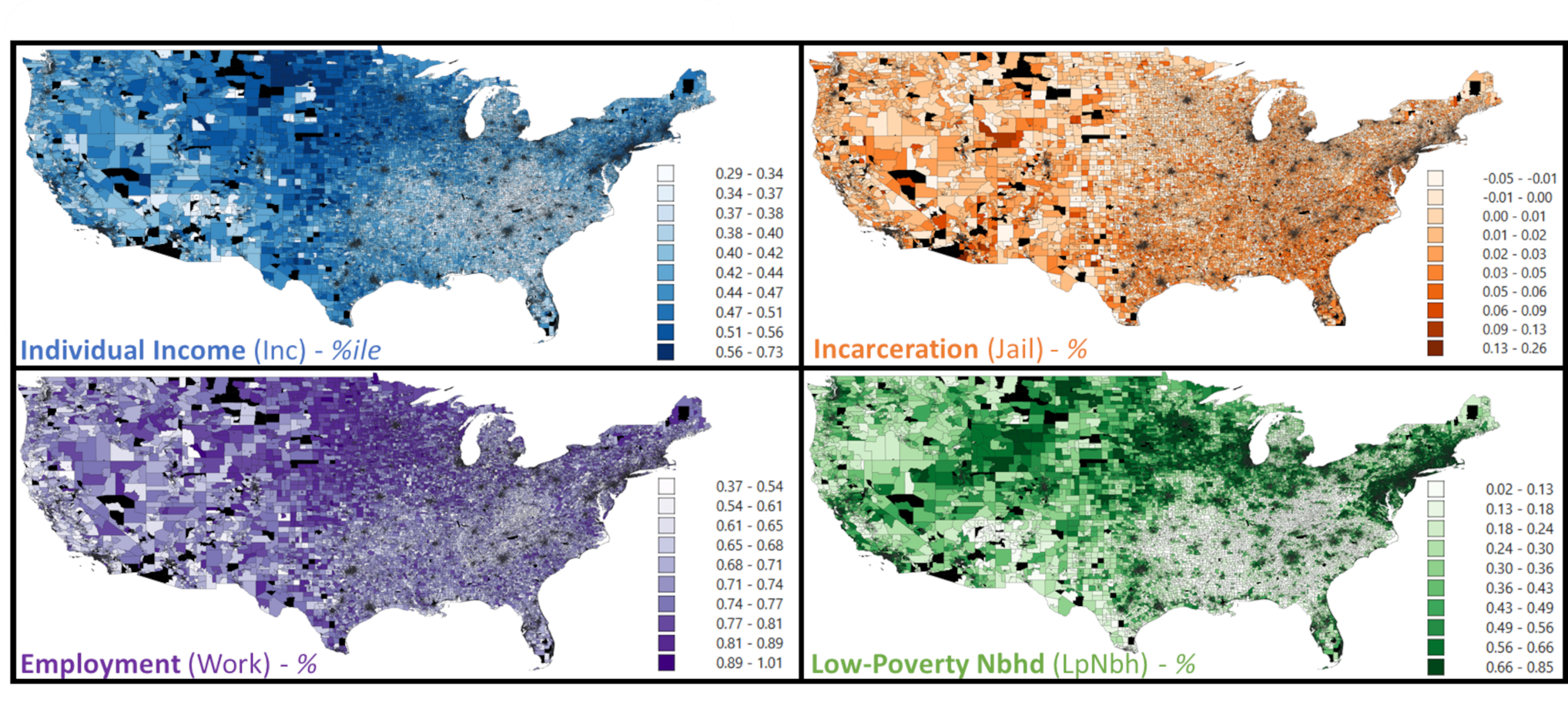


Supplemental Figure S1: Key Variable distributions for individuals who had parental income at the 25th percentile level. Note: some legends denote values <0 or >1. This is due to statistical imputation within the original Opportunity Atlas dataset that we used in our analysis. Inc: individual income measured as percentile of the national income distribution; Jail: incarceration rate measured as percent of residents who are incarcerated; Work: employment measured as percent of residents who are employed; LowPov: low-poverty neighborhood residence measured as percent of residents who live in a neighborhood with <10% poverty

## Supplemental Table S1: Correlations across selected opportunity atlas variables at five parental income levels.

|  | kir1 | kir25 | kir50 | kir75 | kir100 | jal1 | jal25 | jal50 | jal75 | jal100 | wrk1 | wrk25 | wrk50 | wrk75 | wrk100 | lpv1 | lpv25 | lpv50 | lpv75 | lpv100 |
| --- | --- | --- | --- | --- | --- | --- | --- | --- | --- | --- | --- | --- | --- | --- | --- | --- | --- | --- | --- | --- |
| kir1 | 1.0000 | 0.9466 | 0.7518 | 0.3846 | -0.1475 | -0.3096 | -0.3456 | -0.2838 | -0.1413 | -0.0365 | 0.3849 | 0.3571 | 0.1701 | -0.0483 | 0.1179 | 0.5591 | 0.5529 | 0.5232 | 0.4639 | 0.3788 |
| kir25 | 0.9466 | 1.0000 | 0.9242 | 0.6617 | 0.1792 | -0.3304 | -0.3935 | -0.3620 | -0.2176 | -0.1015 | 0.3133 | 0.3755 | 0.3073 | 0.1368 | 0.2723 | 0.6166 | 0.6181 | 0.5982 | 0.5459 | 0.4633 |
| kir50 | 0.7518 | 0.9242 | 1.0000 | 0.8978 | 0.5413 | -0.3092 | -0.3954 | -0.4043 | -0.2778 | -0.1643 | 0.1848 | 0.3451 | 0.4272 | 0.3370 | 0.4173 | 0.5989 | 0.6094 | 0.6039 | 0.5671 | 0.4991 |
| kir75 | 0.3846 | 0.6617 | 0.8978 | 1.0000 | 0.8562 | -0.2260 | -0.3227 | -0.3765 | -0.2945 | -0.2057 | 0.0017 | 0.2446 | 0.4844 | 0.5040 | 0.5055 | 0.4650 | 0.4838 | 0.4960 | 0.4841 | 0.4458 |
| kir100 | -0.1475 | 0.1792 | 0.5413 | 0.8562 | 1.0000 | -0.0689 | -0.1523 | -0.2446 | -0.2365 | -0.2000 | -0.2136 | 0.0622 | 0.4239 | 0.5671 | 0.4756 | 0.1854 | 0.2090 | 0.2387 | 0.2591 | 0.2657 |
| jal1 | -0.3096 | -0.3304 | -0.3092 | -0.2260 | -0.0689 | 1.0000 | 0.9302 | 0.4683 | -0.0504 | -0.3562 | -0.0529 | -0.0460 | -0.0174 | 0.0128 | -0.0100 | -0.2545 | -0.2492 | -0.2320 | -0.2013 | -0.1593 |
| jal25 | -0.3456 | -0.3935 | -0.3954 | -0.3227 | -0.1523 | 0.9302 | 1.0000 | 0.7600 | 0.3197 | 0.0117 | -0.0447 | -0.0618 | -0.0602 | -0.0366 | -0.0560 | -0.3093 | -0.3071 | -0.2925 | -0.2617 | -0.2162 |
| jal50 | -0.2838 | -0.3620 | -0.4043 | -0.3765 | -0.2446 | 0.4683 | 0.7600 | 1.0000 | 0.8588 | 0.6588 | -0.0141 | -0.0673 | -0.1142 | -0.1108 | -0.1170 | -0.2939 | -0.2979 | -0.2933 | -0.2734 | -0.2384 |
| jal75 | -0.1413 | -0.2176 | -0.2778 | -0.2945 | -0.2365 | -0.0504 | 0.3197 | 0.8588 | 1.0000 | 0.9512 | 0.0148 | -0.0494 | -0.1190 | -0.1327 | -0.1265 | -0.1847 | -0.1922 | -0.1970 | -0.1923 | -0.1771 |
| jal100 | -0.0365 | -0.1015 | -0.1643 | -0.2057 | -0.2000 | -0.3562 | 0.0117 | 0.6588 | 0.9512 | 1.0000 | 0.0301 | -0.0320 | -0.1060 | -0.1281 | -0.1153 | -0.0942 | -0.1028 | -0.1126 | -0.1177 | -0.1165 |
| wrk1 | 0.3849 | 0.3133 | 0.1848 | 0.0017 | -0.2136 | -0.0529 | -0.0447 | -0.0141 | 0.0148 | 0.0301 | 1.0000 | 0.8825 | 0.3520 | -0.2192 | 0.2125 | 0.0551 | 0.0551 | 0.0532 | 0.0484 | 0.0408 |
| wrk25 | 0.3571 | 0.3755 | 0.3451 | 0.2446 | 0.0622 | -0.0460 | -0.0618 | -0.0673 | -0.0494 | -0.0320 | 0.8825 | 1.0000 | 0.7508 | 0.2654 | 0.6470 | 0.1085 | 0.1139 | 0.1184 | 0.1173 | 0.1098 |
| wrk50 | 0.1701 | 0.3073 | 0.4272 | 0.4844 | 0.4239 | -0.0174 | -0.0602 | -0.1142 | -0.1190 | -0.1060 | 0.3520 | 0.7508 | 1.0000 | 0.8361 | 0.9894 | 0.1385 | 0.1493 | 0.1610 | 0.1656 | 0.1612 |
| wrk75 | -0.0483 | 0.1368 | 0.3370 | 0.5040 | 0.5671 | 0.0128 | -0.0366 | -0.1108 | -0.1327 | -0.1281 | -0.2192 | 0.2654 | 0.8361 | 1.0000 | 0.9068 | 0.1121 | 0.1234 | 0.1367 | 0.1443 | 0.1441 |
| wrk100 | 0.1179 | 0.2723 | 0.4173 | 0.5055 | 0.4756 | -0.0100 | -0.0560 | -0.1170 | -0.1265 | -0.1153 | 0.2125 | 0.6470 | 0.9894 | 0.9068 | 1.0000 | 0.1361 | 0.1474 | 0.1598 | 0.1654 | 0.1620 |
| lpv1 | 0.5591 | 0.6166 | 0.5989 | 0.4650 | 0.1854 | -0.2545 | -0.3093 | -0.2939 | -0.1847 | -0.0942 | 0.0551 | 0.1085 | 0.1385 | 0.1121 | 0.1361 | 1.0000 | 0.9911 | 0.9415 | 0.8390 | 0.6899 |
| lpv25 | 0.5529 | 0.6181 | 0.6094 | 0.4838 | 0.2090 | -0.2492 | -0.3071 | -0.2979 | -0.1922 | -0.1028 | 0.0551 | 0.1139 | 0.1493 | 0.1234 | 0.1474 | 0.9911 | 1.0000 | 0.9779 | 0.9039 | 0.7799 |
| lpv50 | 0.5232 | 0.5982 | 0.6039 | 0.4960 | 0.2387 | -0.2320 | -0.2925 | -0.2933 | -0.1970 | -0.1126 | 0.0532 | 0.1184 | 0.1610 | 0.1367 | 0.1598 | 0.9415 | 0.9779 | 1.0000 | 0.9733 | 0.8935 |
| lpv75 | 0.4639 | 0.5459 | 0.5671 | 0.4841 | 0.2591 | -0.2013 | -0.2617 | -0.2734 | -0.1923 | -0.1177 | 0.0484 | 0.1173 | 0.1656 | 0.1443 | 0.1654 | 0.8390 | 0.9039 | 0.9733 | 1.0000 | 0.9727 |
| lpv100 | 0.3788 | 0.4633 | 0.4991 | 0.4458 | 0.2657 | -0.1593 | -0.2162 | -0.2384 | -0.1771 | -0.1165 | 0.0408 | 0.1098 | 0.1612 | 0.1441 | 0.1620 | 0.6899 | 0.7799 | 0.8935 | 0.9727 | 1.0000 |

kir: individual income measured as percentile of the national income distribution; jail: incarceration rate measured as percent of residents who are incarcerated; wrk: employment measured as percent of residents who are employed; lpv: low-poverty neighborhood residence measured as percent of residents who live in a neighborhood with <10% poverty. Parental income percentiles (1, 25, 50, 75, 100) designated by number following variable name.


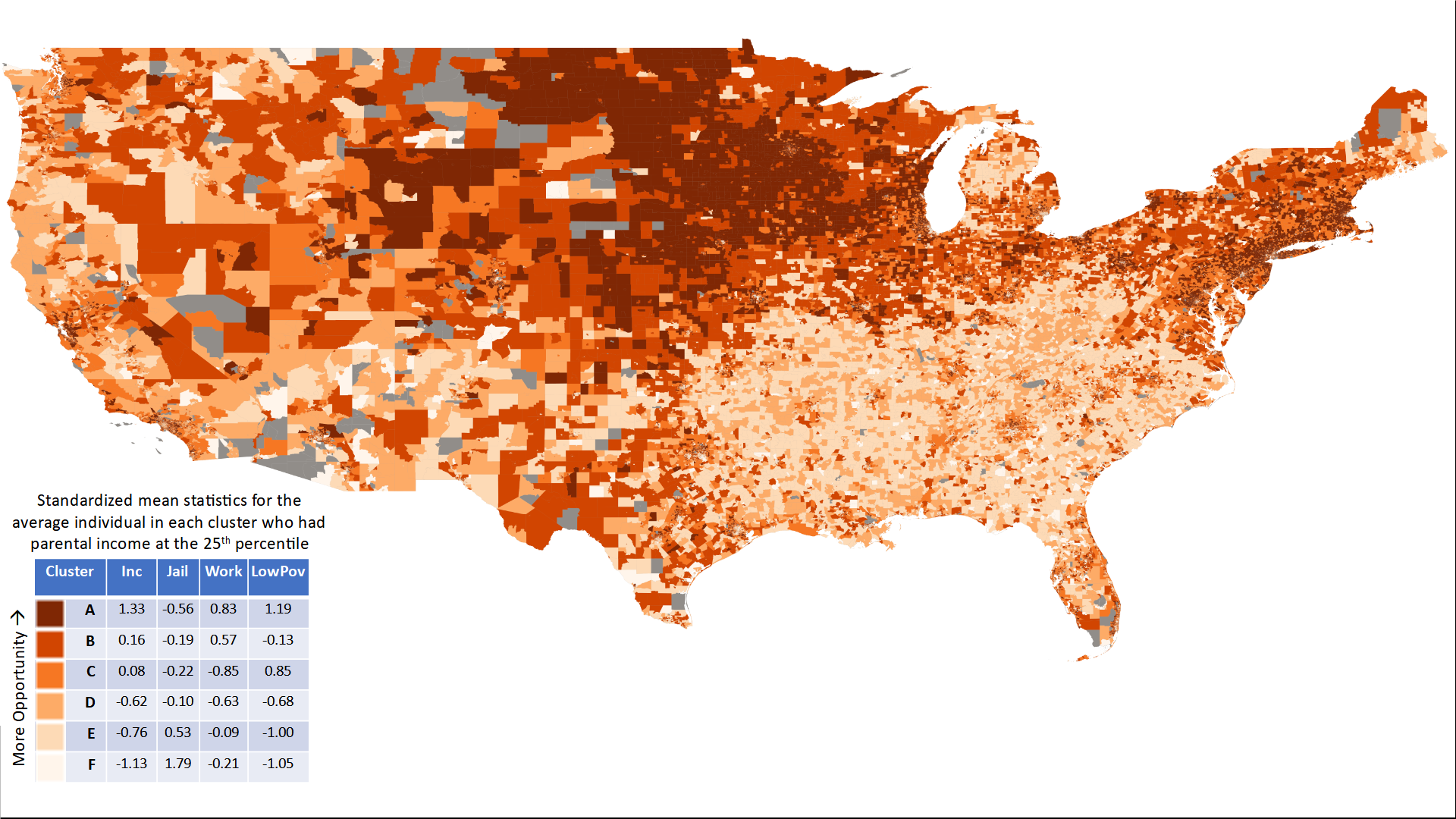
Supplemental Figure S2. Sensitivity Analysis: Six US-wide clusters from standardized Opportunity Atlas variables**.**

When comparing the 6 standardized clusters in this figure to 7 unstandardized clusters in Figure 3, no major differences in cluster spatial trends were observable. *Note: Standardized statistics represent the mean outcomes for individuals who had parental income at the 25^th^ percentile.*


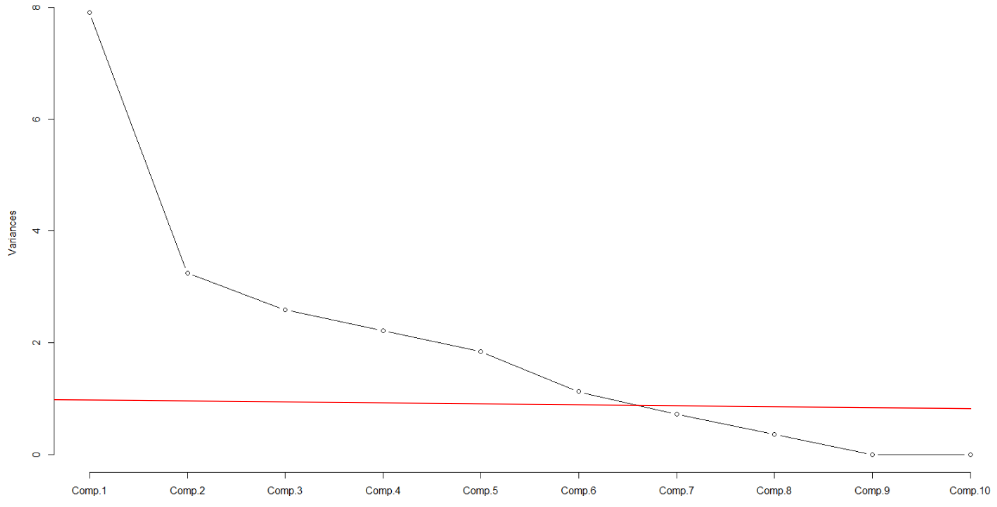


## Supplemental Figure S3: Screeplot of variance of the dataset explained by each component in PCA.

From this Screeplot and numerical summary of the components, six components were retained with eigenvalues of >1; additionally, the cumulative variance explained by all six components was >90%.


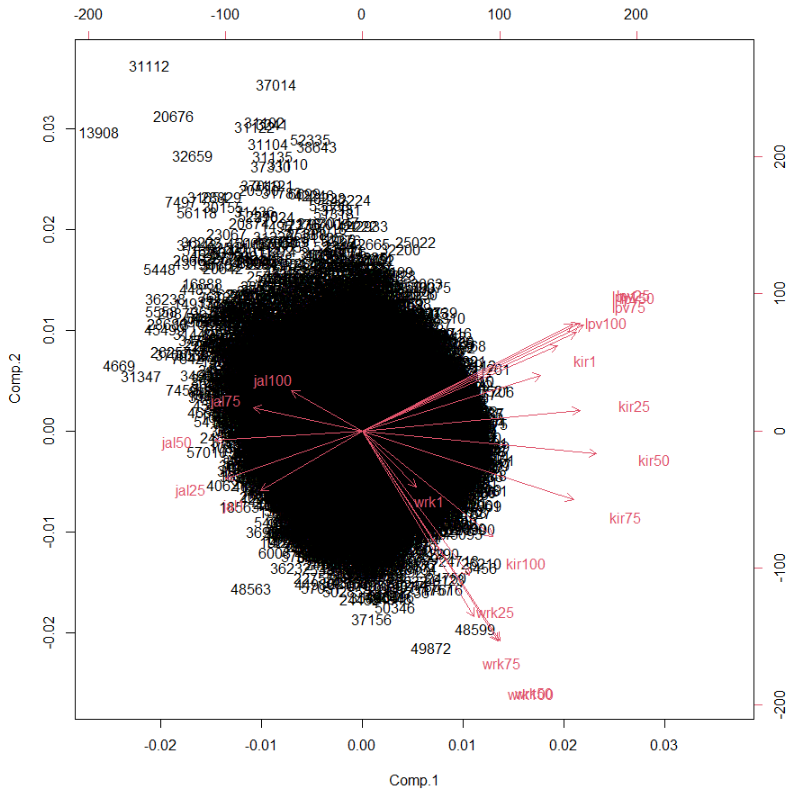


## Supplemental Figure S4: Biplot of each variable and its relationship to Components 1 & 2 during PCA.

The Bi Plot confirms that Income (denoted here by kir) and Low-Poverty Neighborhoods (denoted here by lpv) have similar impacts on the variance while Jail and Work are more independent.


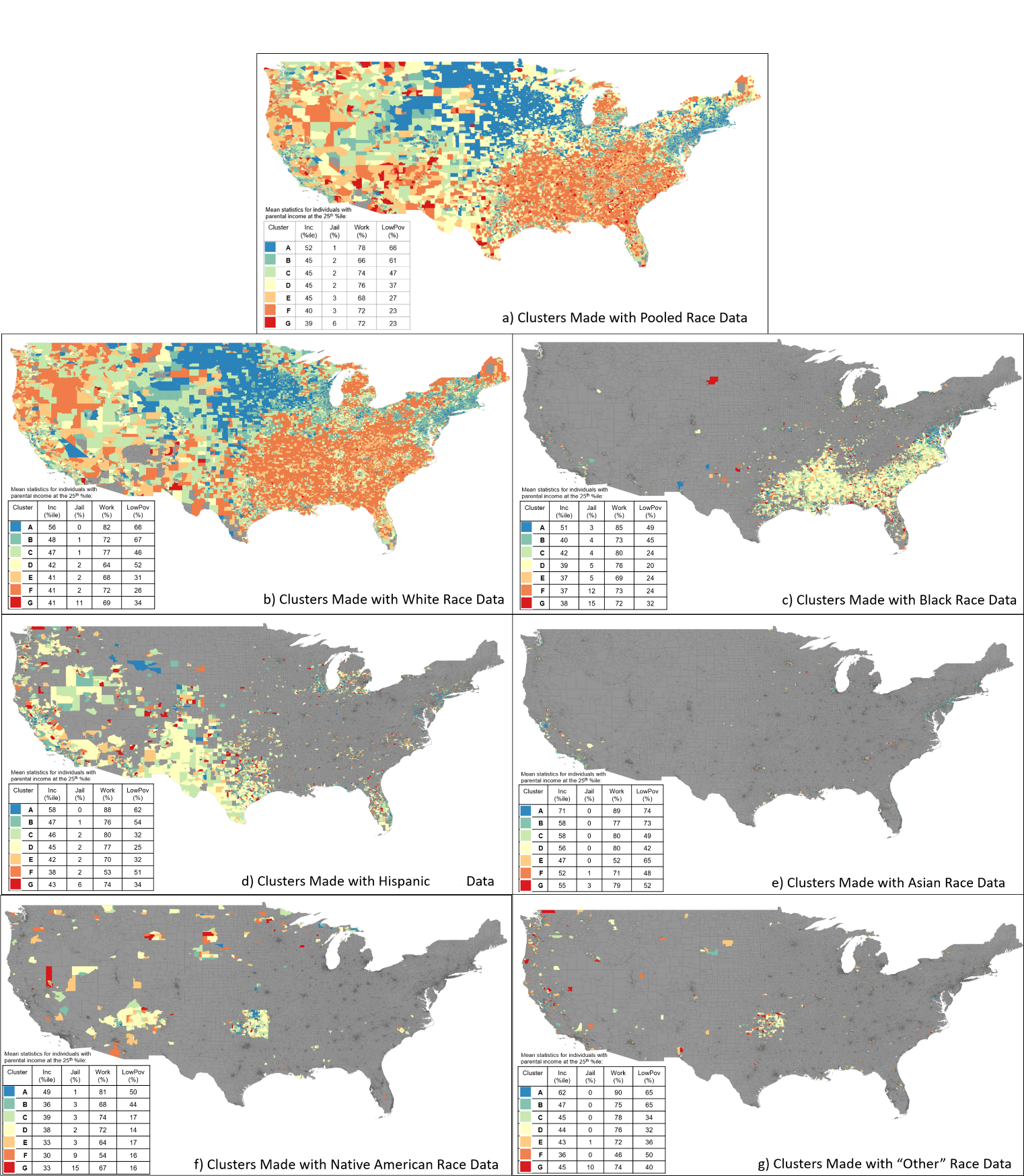


Supplemental Figure S5. Race/ethnicity-specific clusterings derived from the four Opportunity Atlas key variables at five parental income levels. The spatial distribution of clusters nationwide that considers data from all races (Panel S5a) looks very similar to the spatial distribution of clusters made with only data from people who identified as Non-Hispanic Whites (Panel S5b). This is likely due to the fact that most US census tracts have predominately White populations. Figures S5c-g show what the clusters would look like if we were to solely consider data from each non-White race. For each figure, only census tracts with data for that particular race were assigned to a cluster and mapped. Each figure also has its own table that presents mean statistics for each of the derived clusters. Due to the obvious differences in clustering based on race-specific data, we can conclude that the nationwide opportunity clusters presented in this paper might not well represent opportunity levels for Non-White populations. Additionally, it can be seen in this figure that data for each race/ethnicity is sparse nationwide.


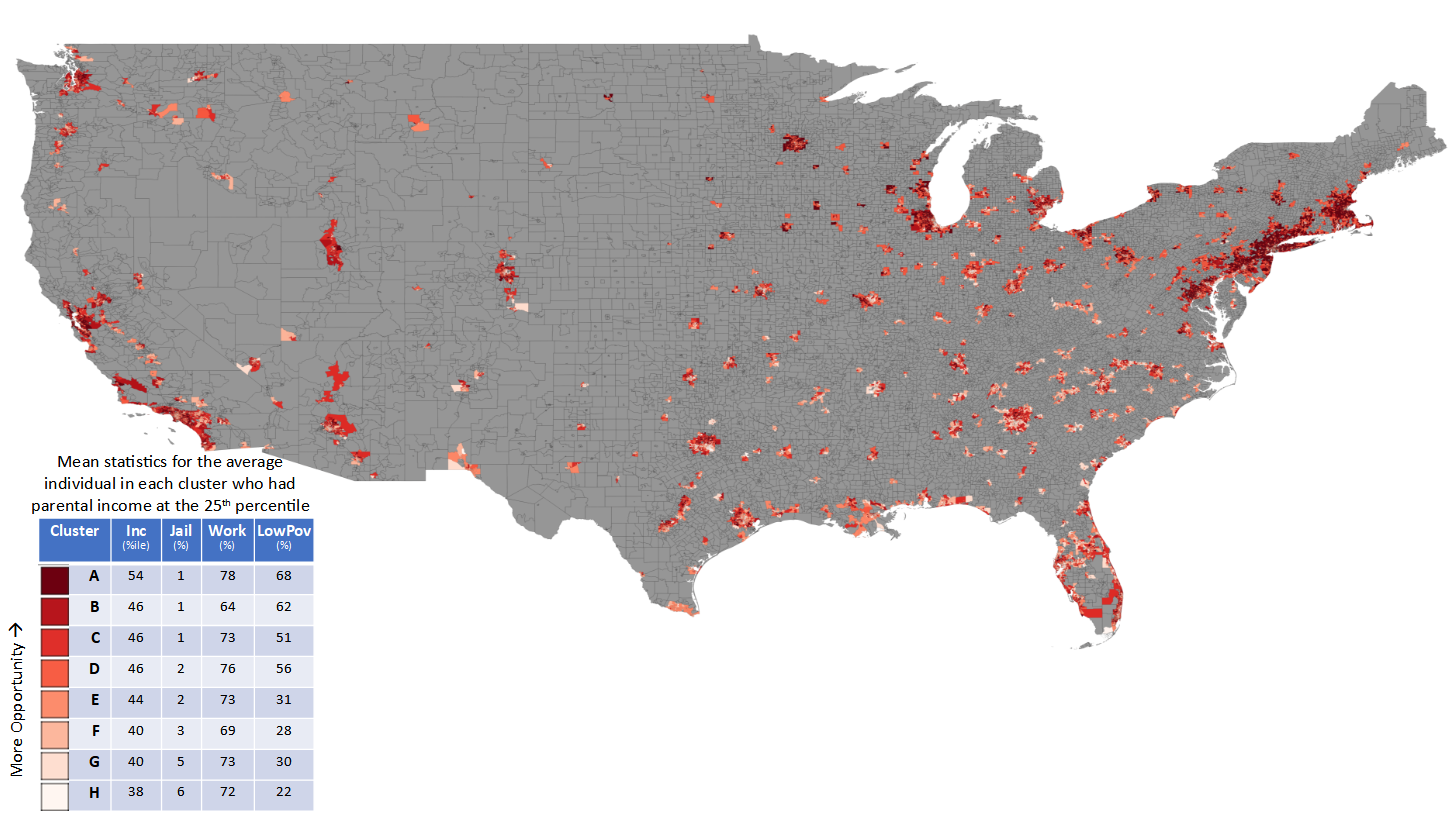


Supplemental Figure S6. US-wide clusters among RUCA 1-designated census tracts and their corresponding statistics derived from the Opportunity Atlas key variables. *Note: Statistics and standardized statistics represent the mean outcomes for individuals who had parental income at the 25^th^ percentile; the clusters presented in the map are derived from all four key variables at all five parental income levels for census tracts with a RUCA 1 designation.*
